# A collection of the etiological theories, characteristics, and observations/phenomena of peptic ulcers in existing data

**DOI:** 10.1016/j.dib.2018.05.022

**Published:** 2018-05-16

**Authors:** Simon X.M. Dong, Connie C.Y. Chang, Katelynn J. Rowe

**Affiliations:** International Institute of Consciousness Science, Ottawa, Ontario, Canada K2K2K3

**Keywords:** Peptic ulcers, Etiological theory, Characteristics, Observations, Phenomena, *Helicobacter pylori*

## Abstract

In this article, we compiled 13 etiological theories, 15 characteristics, and 81 observations/phenomena of peptic ulcers, reported in reproducible, peer-reviewed studies from the literature, to reflect the historical evolution of studies on peptic ulcers and to provide a multidisciplinary view of this disease. This data was collected during the systematic review of topics on peptic ulcers including genetics, etiology, epidemiology, psychology, anatomy, neurology, bacteriology, pathology, and clinical statistics. The data curated herein was extracted via application of recently published basic theories and methodologies.

**Specifications Table**

**Table:** 

Subject area	Medicine
More specific subject area	Gastroenterology
Type of data	Tables
How data was acquired	Systematic review of the existing data over the past 300 years
Data format	Filtered and classified
Data source location	Ottawa, Ontario, Canada
Experimental factors	Not applicable
Experimental features	Not applicable
Data accessibility	Data provided in the article is accessible to the public
Related research articles	6 prepared, unpublished manuscripts on the pathogenesis of peptic ulcers

**Value of the data**

• Etiological theories proposing the correct cause of peptic ulcers should be able to explain all 15 characteristics and 81 observations/phenomena listed herein.
• Many etiological theories summarized herein were supported by valid laboratory results, clinical observations, and/or epidemiological surveys, and did make important discoveries.
• This article may prove useful in advancing the development of new experimental avenues by presenting data together that otherwise might be kept separate.
• Similar literature review can be repeated for any disease. Accordingly, the data obtained can be used to challenge etiological theories and provide new insights for any disease.

## 1 Data

Over the past 300 years, many etiological theories have been proposed to explain the pathogenesis of peptic ulcers (including gastric ulcer and duodenal ulcer), but none of these theories have ever been able to explain all the characteristics and observations/phenomena of this disease [1]. Currently, it is widely believed that there is a causal relationship between *Helicobacter pylori* *(H. pylori)* and peptic ulcers due to the revolutionary discovery of *H. pylori* in 1982 [2]. However, the role of *H. pylori* in peptic ulcers is controversial, and how the bacterial infection can lead to ulceration is presently unknown [3], [4], [5], [6]. To address these challenges, we systematically reviewed all the topics on peptic ulcers over the past 300 years and applied novel basic theories and methodologies to analyze the existing data. We summarized our results into 6 manuscripts (prepared, unpublished) to explain the pathogenesis of peptic ulcers. This article is a byproduct of our systematic review project and provides essential background information for all 6 manuscripts. Our extensive and in-depth literature review indicated that there were 13 etiological theories in history attempting to explain the pathogenesis of peptic ulcers (Table 1). We also found that the pathogenesis of peptic ulcers included 15 characteristics (Table 2) and 81 observations/phenomena, which were grouped into four tables based on diseases and *H. pylori* (Table 3, Table 4, Table 5, Table 6).

**Table 1:** Etiological theories in history.

		**Etiological theory**	**Founder & year**	**Key points**

	1	Circulation Theory [7]	John Hunter, 1772	Gastric acid is neutralized by the continuing circulation of alkaline blood through the tissue.
	2^a^	Ischemia Theory [8]	Rudolf Virchow, 1853	The presence or absence in the gastric mucosa of end arteries whose spasm or thrombosis might account for localized ulceration.
	3	Digestion Theory [9]	Heinrich Irenaeus Quincke, 1882	Peptic ulcer is caused by the proteolytic effects of pepsin and the corrosive effects of gastric acid.
	4	No Acid, No Ulcer [10]	Dragutin (Carl) Schwartz, 1910	Hypersecretion of gastric acid is the cause of peptic ulcer.
	5	Nerve Theory [11]	Von Bergmann G., 1913	The abnormality of neurotransmitters in the central nervous system is the cause of peptic ulcer.
	6	Funktionell-mechanische Theorie [12]	Ludwig Aschoff, 1918	Rubbing of food as it passes through the narrow pyloric portion of the stomach results in peptic ulcer.
	7	Inflammation Theory [13]	Georg Ernst Konjetzny, 1923	Chronic gastritis and duodenitis cause gastric and duodenal ulcers.
	8	Psychosomatics Theory [14]	Franz Gabriel Alexander, 1943	Social, psychological, and behavioral factors are the cause of peptic ulcer.
	9	Stress Theory [15]	Hans Selye, 1950	Stress induced by personality traits, and social and natural events is the cause of peptic ulcer.
	10	Balance Theory [16]	Shay H and Sun C.H., 1963	Peptic ulcer is the result of an imbalance of defensive and aggressive factors in the upper gastrointestinal tract.
	11	Pallium-viscus Theory (The Corticovisceral Theory) [17]	K.M. Bykov and I.T. Kurtsin, 1966	A disturbance in the excitatory and inhibitory processes in the cerebral cortex is the cause of peptic ulcer.
	12	Double Restriction Mechanism [18]	Minoru Oi (大井実, Japanese), 1966	Peptic ulcer is the coefficient results of anatomic factors and functional factors.
	13^b^	Theory of *H. Pylori*[19]	Barry J. Marshal and J. Robin Warren, 1988	Peptic ulcer is an infectious disease caused by the infection of *H. pylori*.

a we designate the etiological theory proposed by Rudolf Virchow as Ischemia Theory according to the mechanism described.

b we designate the etiological theory based on *H. pylori* infection as Theory of *H. pylori*.

**Table 2:** Characteristics of peptic ulcers.

	**Classification**	**Characteristics**	Year

	General (3)	1) Genetic predisposition [20]	1967
		2) Etiology [21]	1986
		3) Epidemiology [22]	1984
	Clinical symptoms (6)	4) Predilection sites [23]	2009
		5) Morphology [24]	2004
		6) Bleeding [25]	2010
		7) Perforation [23]	2009
		8) Relapse [26]	1998
		9) Multiplicity [27]	2002
	Local aggressive factors in the upper digestive tract (3)	10) Gastric acid and pepsin [10]	1910
		11) *Helicobacter pylori*[19]	1988
		12) NSAIDs and other medications [27]	2002
	Prognosis (3)	13) Self-healing [28]	1951
		14) Effects of clinical treatments [29]	1995
		15) Hospitalization rates, morbidity, and mortality [22]	1984

**Table 3:** Duodenal ulcer-related observations/phenomena.

	**Observations/phenomena**	**Year**

	1. No Acid, No Ulcer (*true statement for duodenal ulcer*) [10], [24].	1910
		2004
	2. The role of gastric acid in the pathogenesis of duodenal ulcer is further supported by the relief of pain observed after neutralization or buffering of gastric contents with alkali or food [24], [30].	1978
		2004
	3. Doll and Jones’ survey suggested a positive correlation between stressful occupations and duodenal ulcer, and a decreased incidence of ulcer among agricultural workers [31], [32].	1951
		1952
	4. Studies suggest that severe anxiety caused acid hypersecretion which, in turn, contributed to ulceration and symptoms. The fact that acid hypersecretion and symptoms abated with alleviation of stress supports this hypothesis [33].	1983
	5. Rates of recurrence in patients whose initial ulcers healed during conventional anti-secretory therapy range from 60 to 100 percent per year [29].	1995
	6. Duodenal ulcer had higher incidence in large cities compared to rural areas in Africa since the 1950s [34].	1995

**Table 4:** Gastric ulcer-related observations/phenomena.

	**Observations/phenomena**	**Year**

	7. No Acid, No Ulcer (*incorrect for gastric ulcers*) [10], [24].	1910
		2004
	8. In contrast to patients with duodenal ulcer, most patients with gastric ulcer are normosecretors or hyposecretors. Decreased acid-peptic activity in these patients suggests impaired mucosal defence [10], [35].	1996
		2011
	9. Gastric ulcer is a sharply circumscribed loss of tissue involving the mucosa, submucosa, and muscular layer. Gastric ulcer has a characteristic “punch out” appearance with clean edges, as if it were cut by a knife [20], [36].	1967
		2007
	10. Gastric ulcers can be induced in only 8–30% of mouse models [37].	1991
	11. Gastric ulceration begins in the mucosa and extends into the wall of the stomach [31].	1952
	12. Bleeding and perforation of gastric ulcers [33], [38].	1983
		1991
	13. Self-healing and effects of clinical therapy [39], [40].	1998
		2004
	14. Stress-related gastric lesions are ‘brain-driven’ events that may be more effectively managed through central manipulations than by altering local, gastric factors. For example, stimulation or lesions of the central nucleus of the amygdala produced or reduced gastric ulcers, respectively [37], [41], [42].	1980
		1991
		1998
	15. Development of gastric ulcers elicited by cold stress was significantly decreased by i.p. pre-treatment with EDTA or a-methyl tyrosine, which depleted neurotransmitters. Gastric ulcers were significantly increased by pre-treatment with CaCl_2_[43].	1998
	16. The predilection sites of gastric ulcers are the gastric antrum and lesser curvature [23].	2009
	17. Vulnerability to gastric ulceration is modulated by psychologically meaningful experiences. Repeated stress of the same type generally, but not exclusively, provides some degree of protection against ulcer during the second or later exposures [37], [44].	1991
		2000

**Table 5:** Both gastric and duodenal ulcer-related observations/phenomena.

	**Observations/phenomena**	**Year**

	18. Birth-cohort Phenomenon: the mortality rate of gastric ulcers in England and Wales increased at the beginning of the 20th century, reached a peak and then began to fall in the early 1950s. Similar trends were found for duodenal ulcers, but followed approximately five years behind [45], [46].	1962
		2006
	19. Once an ulcer, always an ulcer [47].	1994
	20. Seasonal occurrence of peptic ulcer diseases [48], [49].	1984
		1994
	21. Patients free of ulcer distress for long periods of time were subjected to emotional trauma and feelings of insecurity during the symptom-free intervals [31].	1952
	22. Investigations of the effects of perceived stress on physiological parameters are scarce and the findings are often conflicting [50].	2005
	23. There is no definitive study proving a causal relationship between psychological stress and the development of ulcer disease [51].	2006
	24. Feldman's multidimensional case-controlled study found that ulcer patients exhibited significantly more emotional distress in the form of depression and anxiety. Hypochondriasis, a negative perception of their life events, dependency, and lowered self-confidence were the four variables that best discriminated ulcer patients from controls [52].	1986
	25. Peptic ulcer is a rare disease in childhood [53], [54].	1961
		2010
	26. Although gastric ulcer and duodenal ulcer share something in common, they are believed to be different diseases [20], [55].	1967
		2002
	27. The final stage of ulceration is a corrosive rather than an infectious process [31], [56].	1945
		1952
	28. The gastric acid secretion of duodenal ulcer patients is much higher than normal controls, but only 7–8.5% of the duodenal ulcer patients suffer from gastric ulcer simultaneously [57], [58].	1999
		2004
	29. Severe emotional stress may contribute to ulcer perforation and bleeding in some patients [33].	1983
	30. Many uncomplicated lesions heal in spite of the presence of acid gastric content, as shown by the “spontaneous” remissions of the disease and by the healed scars found at x-ray and at autopsy; however, the healing of peptic ulcer is much more rapid when the lesion is protected from the action of acid gastric juice [31].	1952
	31. Autopsy reports showed: 20%-29% of males and 11%-18% of females were found to have suffered from ulcers in the past or present [59], [60].	1960
		1978
	32. It is believed that, not only should the prognosis and assessment of ulcer have mental assessment, but the treatment without mind adjustment is also incomplete [21].	1986
	33. Peptic ulcer patients may have “ulcer personality”, such as immaturity, impulsivity, feelings of social isolation, and alienation [52].	1986
	34. In a 2-year study of Pima Indians, Hesse did not find any peptic ulcer disease [61].	1959
	35. In contrast to Pima Indians, 10% of Caucasians develop peptic ulcers [62], [63].	1955
		1962
	36. To date, no consistent pattern of factors, in either host or organism, has been identified that successfully predicts which infected persons will subsequently have ulcer disease [64].	1990
	37. The relationship between life event stresses, psychological factors and peptic ulcer diseases is not clearly established at the present time and warrants further study [52].	1986
	38. Richard emphasized the different aetiology of gastric and duodenal ulcers; persons with gastric and duodenal ulcers differ epidemiologically, behaviourally, and genetically [20], [55].	1967
		2002
	39. Gastric ulcer was more frequent than duodenal ulcer, 4 gastric:1 duodenal in 1900 versus 1 gastric:10 duodenal currently. More women than men had the disease (3F:1M), but now it has become reversed; as the ratio for gastric ulcer is now 1F:4M and 1F:10M for duodenal ulcer [20], [65].	1953
		1967
	40. Stress ulcers in the rat are primarily gastric rather than duodenal, the latter typically requiring additional artificial chemical potentiation (e.g., histamine) [37].	1991
	41. Many ulcer patients and some physicians believe that symptomatic exacerbations of peptic ulcer disease occur during or shortly after stressful events [33], [66], [67].	1973
		1977
		1983
	42. Mental disorders (or stress) are associated with increased rates of peptic ulcer diseases [68], [69].	2009
		2013
	43. The spontaneous remissions and relapses of peptic ulcers have never been explained [31].	1952
	44. The pathophysiology of peptic ulcer has centred on an imbalance between aggressive and protective factors [70].	2004
	45. No single theory in history could fully explain the pathogenesis of peptic ulcers [1].	1990

**Table 6:** *H. pylori*-related observations/phenomena.

	**Observations/phenomena**	**Year**

	46. African Enigma: the *H. pylori* infection rate is high (close to 100%) throughout Africa, but the prevalence of duodenal ulcer varied in different parts of the continent [34].	1995
	47. Only the presence of duodenal ulcers, and not gastric ulcers, was associated with increasing *H. pylori* density. The association between gastric ulcers and *H. pylori* infection is less clear [71].	1992
	48. Only 27% of symptomatic children with peptic ulcers were *H. pylori* positive [72].	2001
	49. 48% of patients developed ulcers within six months of healing, but the re-infection rate after eradication was very low (<2%) [5].	1994
	50. In developing countries with uniformly high prevalence of *H. pylori* infection, there are marked regional differences in the prevalence of duodenal ulcers, which could not be explained by the more toxic CagA and VacA *H. pylori* strains [6].	1999
	51. In the countries with low prevalence of *H. pylori*, 30–40% or more of duodenal ulcer patients are *H. pylori* negative, and the absence of *H. pylori* infection in early cases of duodenal ulcers was also reported [6], [73].	1998
		1999
	52. The role of *H. pylori* in peptic ulcers is controversial [3], [4], [5], [6].	1994
		1995
		1999
		2009
	53. *H. pylori* is the most important aetiological factor so far described for duodenal ulcer [19].	1988
	54. How *H. pylori* infection can lead to ulceration is unknown [74].	2002
	55. No *H. pylori*, No Ulcer; peptic ulcer is an infectious disease [75].	1989
	56. In spite of a high prevalence of *H. pylori* infection worldwide, the incidence of duodenal ulcer disease in both adults and children is low in comparison [72], [76], [77], [78].	1987
		1988
		1991
		2001
	57. Kato and colleagues’ retrospective analysis found that *H. pylori* prevalence in gastric ulcer did not reach 50%; they concluded while *H. pylori* infection appears to be a risk factor in gastric ulcer, other causes are responsible for most cases. Only 56–96% of gastric ulcer patients are *H. pylori* positive, so other factors must be involved [34], [79].	1995
		2004
	60. There are basically three different types of peptic ulcer: *H. pylori*-related peptic ulcer; NSAID-related peptic ulcer; and Non-*H. pylori*, non-NSAID ulcer [70].	2004
	61. A relatively isolated group of Australian aboriginals have virtually no *H. pylori* infection and hardly any peptic ulcer disease [34], [80].	1995
		1976
	62. Up to 20% of patients with ulcers suffer a relapse of ulcer disease despite successful eradication of their infections, suggesting that *H. pylori* was not the cause of their original ulcers [73], [81].	1998
		2001
	63. Difference in virulence of *H. pylori* strains (cag- and cag+) has been considered as a putative explanation as to why only a minority of infected population develop peptic ulcers [82], [83], [84].	1996
		1998
		2002
	64. *H. pylori* infection in rats was successful and was accompanied by a mild to moderate mucosal inflammation. After *H. pylori* inoculation, an ulcer was induced in the oxyntic mucosa of both infected and uninfected rats by exposing the serosal side to acetic acid [85].	1998
	65. More than 95% of patients with duodenal ulcers and more than 80% of patients with gastric ulcers are infected with *H. pylori*[29], [86], [87], [88].	1991
		1994
		1995
	66. The corresponding ulcer areas in the *H. pylori*-infected rats were significantly larger in the infected than in the uninfected rats, and ulcer healing was delayed in the infected rats. Eliminating *H. pylori* accelerates the healing of ulcer [85], [89], [90], [91].	1992
		1997
		1998
		2003
	67. Eradication of *H. pylori* in gastric ulcer patients has also been shown to be associated with a significant reduction in ulcer relapse rate, compared with those who remain infected [29], [34].	1995
	68. Clinical data reported that the recurrence rate is as high as 74–80% in *H. pylori* positive group of duodenal ulcer patients who have healed, but the negative group is only 0–28%. The discrimination was remarkable [57].	1999
	69. A negative interaction between *H. pylori* and NSAIDs on duodenal ulcers suggests that *H. pylori* reduces the development of ulcers in NSAIDs users [34].	1995
	70. ~20% of peptic ulcers in the Polish population are unrelated to *H. pylori* and NSAIDs use (idiopathic ulcers) [90].	1997
	71. The prevalence of *H. pylori* in patients with bleeding ulcers may be 15–20% lower than in patients with non-bleeding ulcers [38], [89], [90], [92].	1992
		1997
		2003
	72. The eradication of *H. pylori* reduces the rate of re-bleeding in patients with ulcer disease [90], [93], [94].	1993
		1994
		1997
	73. How *H. pylori* infection affects gastric acid secretion is still unclear.[95]	1998
	74. The incidence of peptic ulcer was higher in *H. pylori* infected patients than in the *H. pylori* negative group [96].	1999
	**Observations/phenomena**	**Year**

	75. Duodenal acid load determines whether *H. pylori* can cause duodenal ulcer [83].	1998
	76. The increase in *H. pylori* density is related to the presence of duodenal ulcer disease [83], [97].	1998
		1999
	77. *H. pylori*-negative duodenal ulcers were associated with a poorer prognosis mainly because of a higher rate of ulcer and symptom relapse [27], [98], [99].	2000
		2001
		2002
	78. When *H. pylori* persisted, 61% of duodenal ulcers healed and 84% relapsed. When H pylori was cleared 92% of ulcers healed and only 21% relapsed during the 12 months follow-up period [19].	1988
	79. Jyotheeswaran and colleagues from greater Rochester, New York, reported a 48% prevalence of *H. pylori* -negative duodenal ulcers in white patients and 15% in non-white patients, with an overall negative prevalence of 39%. Parsonnet's meta-analysis indicates the overall prevalence of *H. pylori*-negative duodenal ulcers is 40% [26], [100].	1998
	80. A review by van der Voort and colleagues suggests that existing data are consistent with a causal role for *H. pylori* in stress ulcer formation [101].	2001
	81. Barry Marshall drank a concoction made from cultured *H. pylori* and came down with gastritis that could be cured with antibiotics [102].	1984

## 2 Materials and methods

We developed a four-step strategy to collect the data presented herein. First, we reviewed all the topics on peptic ulcers, such as genetics, etiology, epidemiology, psychology, anatomy, neurology, bacteriology, pathology, and clinical statistics. To achieve this goal, we searched Medline, Embase, Web of Science, and Google Scholar for articles published over the past 300 years. Books published on peptic ulcers and on the pathogenesis of human disease were also included. Our search was completed without language restrictions. Second, we extracted data on study year, etiological theories, characteristics, observations, and phenomena of peptic ulcers. If an etiological theory in history had yet to be officially named, we named it based on its central idea. For instance, we designate the etiological theory based on *H. pylori* infection as “Theory of *H. pylori*”. Third, selected studies were summarized and unreproducible studies were excluded. If several studies had similar findings, we randomly selected one or two to avoid repetitive results. However, if a paper was identified to be the earliest study on a characteristic or phenomenon, this paper was selected because we determined the earliest paper provided the basis for the other similar studies that followed. Fourth, selected data was classified and curated into 6 tables.
